# Supplementary material for: How does the dental benefits act encourage Australian families to seek and utilise oral health services?
Source: PLoS One. 2022 Nov 28;17(11):e0277152. doi: 10.1371/journal.pone.0277152 (PMC9704637; doi:10.1371/journal.pone.0277152)
Supplement: Attachment — Submitted filename: PONE-D-22-22909 PR.pdf [file pone.0277152.s001.pdf]

PLOS ONE

HOW DOES THE DENTAL BENEFITS ACT ENCOURAGE AUSTRALIAN FAMILIES TO SEEK AND UTILISE ORAL HEALTH SERVICES?

--Manuscript Draft--

**Manuscript Number:**	PONE-D-22-22909
**Article Type:**	Research Article
**Full Title:**	HOW DOES THE DENTAL BENEFITS ACT ENCOURAGE AUSTRALIAN FAMILIES TO SEEK AND UTILISE ORAL HEALTH SERVICES?
**Short Title:**	Utilising oral health services by Australians and the role of the policies
**Corresponding Author:**	Peivand Bastani<br>The University of Queensland<br>Brisbane, QLD AUSTRALIA
**Keywords:**	Dental Benefits Act, Oral health, Dental care utilisation, Qualitative document analysis
**Abstract:**	Background: This study aimed to analyse the content of the Dental Benefits Act 2008 as a foundation for the Child Dental Benefits Schedule (CDBS) to determine how the Act encourages Australian families to seek and utilise oral health services.<br>Methods: This was a qualitative narrative document analysis conducted in 2022. Data was collected by searching formal websites for retrieving documents that reported the Australian Dental Benefits Act. The eligibility of the retrieved documents was assessed based on authenticity, credibility, representativeness, and meaningfulness of the data. A seven-steps procedure was applied for framework analysis.<br>Results: The content of the Dental Benefits Act 2008 forms directions on three categories of operational, collective, and constitutional rules. Operational rules at the level of oral health providers and the population as the service end users can be demonstrated as rules in use in a mutual interaction with the collective and constitutional rules. The consequence of governing the rules at the community level can easily define how the oral health services are really provided and utilised. The response is sent to the government level for better regulation of oral health service delivery and utilisation, then, with an interaction and advocacy with the diverse range of stakeholders and interdisciplinary partnerships with the community groups, non-government sectors and councils, the rules can be transformed, adopted, monitored, and enforced. Another mechanism of response has occurred from the providers and users' level and the operational rules to community groups and stakeholders via advertising and promoting the utilisation and provision of oral health services.<br>Conclusion: This study integrates the perspective of politicians with those of policy makers to reconsider the role and significance of the rules based on the triple collaborations among oral health users and oral service providers, the community, and the stakeholders as well as the government. A comprehensive attention is still needed in the future revisions of the Act according to the contextual factors, socioeconomic and geographical attributes of the population for better implementation of de facto rules and more effective outcomes of the interventions. It is recommended that further research be undertaken utilising a mix-method approach for a holistic view prior to further revisions of the Act or proposal of probable upcoming schemes.
**Order of Authors:**	Peivand Bastani
	Reyhane Izadi
	Nithin Manchery
	Diep Ha
	Hanny Calache
	Ajesh George
	Loc Do
**Additional Information:**	
**Question**	**Response**

Powered by Editorial Manager® and ProduXion Manager® from Aries Systems Corporation

**Financial Disclosure**<br><br>Enter a financial disclosure statement that describes the sources of funding for the work included in this submission. Review the submission guidelines for detailed requirements. View published research articles from *PLOS ONE* for specific examples.<br><br>This statement is required for submission and **will appear in the published article** if the submission is accepted. Please make sure it is accurate.<br><br>**Unfunded studies**<br>Enter: *The author(s) received no specific funding for this work.*<br><br>**Funded studies**<br>Enter a statement with the following details:<br>• Initials of the authors who received each award<br>• Grant numbers awarded to each author<br>• The full name of each funder<br>• URL of each funder website<br>• Did the sponsors or funders play any role in the study design, data collection and analysis, decision to publish, or preparation of the manuscript?<br>• **NO** - Include this sentence at the end of your statement: *The funders had no role in study design, data collection and analysis, decision to publish, or preparation of the manuscript.*<br>• **YES** - Specify the role(s) played.<br><br>* typeset	The author(s) received no specific funding for this work.
**Competing Interests**<br><br>Use the instructions below to enter a competing interest statement for this submission. On behalf of all authors, disclose any competing interests that could be perceived to bias this work—acknowledging all financial support and any other relevant financial or non-financial competing interests.<br><br>This statement is required for submission	The authors have declared that no competing interests exist.

and **will appear in the published article** if the submission is accepted. Please make sure it is accurate and that any funding sources listed in your Funding Information later in the submission form are also declared in your Financial Disclosure statement.<br><br>View published research articles from *PLOS ONE* for specific examples.<br><br>**NO authors have competing interests**<br><br>Enter: *The authors have declared that no competing interests exist.*<br><br>**Authors with competing interests**<br><br>Enter competing interest details beginning with this statement:<br><br>*I have read the journal's policy and the authors of this manuscript have the following competing interests: [insert competing interests here]*<br><br>* typeset	
**Ethics Statement**<br><br>Enter an ethics statement for this submission. This statement is required if the study involved:<br><br>• Human participants<br>• Human specimens or tissue<br>• Vertebrate animals or cephalopods<br>• Vertebrate embryos or tissues<br>• Field research<br><br>Write "N/A" if the submission does not require an ethics statement.<br><br>General guidance is provided below. Consult the submission guidelines for detailed instructions. **Make sure that all information entered here is included in the Methods section of the manuscript.**	N/A

**Format for specific study types**<br><br>**Human Subject Research (involving human participants and/or tissue)**<br>• Give the name of the institutional review board or ethics committee that approved the study<br>• Include the approval number and/or a statement indicating approval of this research<br>• Indicate the form of consent obtained (written/oral) or the reason that consent was not obtained (e.g. the data were analyzed anonymously)<br><br>**Animal Research (involving vertebrate animals, embryos or tissues)**<br>• Provide the name of the Institutional Animal Care and Use Committee (IACUC) or other relevant ethics board that reviewed the study protocol, and indicate whether they approved this research or granted a formal waiver of ethical approval<br>• Include an approval number if one was obtained<br>• If the study involved *non-human primates*, add *additional details* about animal welfare and steps taken to ameliorate suffering<br>• If anesthesia, euthanasia, or any kind of animal sacrifice is part of the study, include briefly which substances and/or methods were applied<br><br>**Field Research**<br><br>Include the following details if this study involves the collection of plant, animal, or other materials from a natural setting:<br>• Field permit number<br>• Name of the institution or relevant body that granted permission	
**Data Availability**<br><br>Authors are required to make all data underlying the findings described fully available, without restriction, and from the time of publication. PLOS allows rare exceptions to address legal and ethical concerns. See the PLOS Data Policy and FAQ for detailed information.	Yes - all data are fully available without restriction

A Data Availability Statement describing where the data can be found is required at submission. Your answers to this question constitute the Data Availability Statement and **will be published in the article**, if accepted.<br><br>**Important:** Stating 'data available on request from the author' is not sufficient. If your data are only available upon request, select 'No' for the first question and explain your exceptional situation in the text box.<br><br>Do the authors confirm that all data underlying the findings described in their manuscript are fully available without restriction?	
**Describe where the data may be found in full sentences. If you are copying our sample text, replace any instances of XXX with the appropriate details.**<br><br>• If the data are **held or will be held in a public repository**, include URLs, accession numbers or DOIs. If this information will only be available after acceptance, indicate this by ticking the box below. For example: *All XXX files are available from the XXX database (accession number(s) XXX, XXX.).*<br>• If the data are all contained **within the manuscript and/or Supporting Information files**, enter the following: *All relevant data are within the manuscript and its Supporting Information files.*<br>• If neither of these applies but you are able to provide **details of access elsewhere**, with or without limitations, please do so. For example:<br><br>*Data cannot be shared publicly because of [XXX]. Data are available from the XXX Institutional Data Access / Ethics Committee (contact via XXX) for researchers who meet the criteria for access to confidential data.*<br><br>*The data underlying the results presented in the study are available from (include the name of the third party*	All relevant data are within the manuscript and its Supporting Information files.

and contact information or URL).<br>• This text is appropriate if the data are owned by a third party and authors do not have permission to share the data.<br><br>* typeset	
Additional data availability information:	Tick here if the URLs/accession numbers/DOIs will be available only after acceptance of the manuscript for publication so that we can ensure their inclusion before publication.

Manuscript

Click here to access/download;Manuscript;Final version ACT 2008.docx

HOW DOES THE DENTAL BENEFITS ACT ENCOURAGE AUSTRALIAN FAMILIES TO SEEK AND UTILISE ORAL HEALTH SERVICES?

Authors

Peivand Bastani[1*], Reyhane Izadi[2], Nithin Manchery[1], Diep Ha[1], Hanny Calache[3], Ajesh George[4], Loc Do[1]

1- School of Dentistry, The University of Queensland, Brisbane, Queensland, Australia
2- School of Health Management and Information Sciences, Shiraz University of Medical Sciences, Shiraz, Iran
3- La Trobe Rural Health School, La Trobe University, Australian Centre for Integration of Oral Health (ACIOH)
4- Australian Centre for Integration of Oral Health (ACIOH), School of Nursing and Midwifery, Western Sydney University. Ingham Institute Applied Medical Research. School of Dentistry, The University of Sydney

Corresponding author:

Dr Peivand Bastani
School of Dentistry
The University of Queensland
Herston, Brisbane, QLD 4006
Australia
Email: p.bastani@uq.edu.au

Abstract

Background: This study aimed to analyse the content of the Dental Benefits Act 2008 as a foundation for the Child Dental Benefits Schedule (CDBS) to determine how the Act encourages Australian families to seek and utilise oral health services.
Methods: This was a qualitative narrative document analysis conducted in 2022. Data was collected by searching formal websites for retrieving documents that reported the Australian Dental Benefits Act. The eligibility of the retrieved documents was assessed based on authenticity, credibility, representativeness, and meaningfulness of the data. A seven-steps procedure was applied for framework analysis.
Results: The content of the Dental Benefits Act 2008 forms directions on three categories of operational, collective, and constitutional rules. Operational rules at the level of oral health providers and the population as the service end users can be demonstrated as rules in use in a mutual interaction with the collective and constitutional rules. The consequence of governing the rules at the community level can easily define how the oral health services are really provided and utilised. The response is sent to the government level for better regulation of oral health service delivery and utilisation, then, with an interaction and advocacy with the diverse range of stakeholders and interdisciplinary partnerships with the community groups, non-government sectors and councils, the rules can be transformed, adopted, monitored, and enforced. Another mechanism of response has occurred from the providers and users' level and the operational rules to community groups and stakeholders via advertising and promoting the utilisation and provision of oral health services.
Conclusion: This study integrates the perspective of politicians with those of policy makers to reconsider the role and significance of the rules based on the triple collaborations among oral health users and oral service providers, the community, and the stakeholders as well as the government. A comprehensive attention is still needed in the future revisions of the Act according to the contextual factors, socioeconomic and geographical attributes of the population for better implementation of de facto rules and more effective outcomes of the interventions. It is recommended that further research be undertaken utilising a mix-method approach for a holistic view prior to further revisions of the Act or proposal of probable upcoming schemes.

Keywords: Dental Benefits Act, Oral health, Dental care utilisation, Qualitative document analysis

Background

Oral health is integral to overall health and well-being of the population. This includes physical, psychological, emotional, and social domains (1). Due to the multidimensional impacts of oral diseases, which include chewing problems, nutritional deficiencies and weight loss, irritability, insomnia, low self-esteem, and decline in social confidence and performance (2), oral health is considered, by World Health Organisation (WHO), to be an essential part of general health interventions and practices (3). To achieve this goal, global policymakers try to develop strategies to increase the population`s access to dental services along with improving their oral health literacy, behaviours and utilisation based on their contextual conditions, infrastructures, and facilities (4). For instance, the National Institute for Health and Care Excellence (NICE) released policies and interventions for the improvement of the population`s oral health. These strategies are included in local health and wellbeing policies, public service environment and workforce policies, nursery services and schools' policies, and policies identifying high-risk groups for poor oral health (5).

In Australia, the provision of oral health services is either via a fee for services mechanism that operates within the private sector or via the provision of oral health services through ~~Commonwealth and~~ State and Territory Government public sector for eligible population groups, which includes children and those with low socioeconomic status. Although there is a policy shift toward preventive services and early intervention programs among children and teenagers, two federal dental programs based on ~~a rich background of~~ national laws and legislations were implemented over the period 2007 to 2013. These were the Chronic Disease Dental Scheme (CDDS) from 2007 to 2012 and the Medicare Teen Dental Plan (MTDP) during 2008 to 2013. The evidence related to the implementation of these programs indicated that these programs were not cost-effective with low utilisation rates particularly in rural and remote areas (6).

The Child Dental Benefits Schedule (CDBS) was implemented in 2014 to provide clinical dental treatments to eligible 2-17 years-old children and teenagers up to a certain payment limit (7). Since 2022, the eligibility has been increased to children 0-17 years with a payment limit of $1026.00 over 2 consecutive calendar years. Despite a wider range of inclusion of dental treatment services in CDBS than in MTDP, low utilisation rate (23%) is existing based on some available evidence as about a quarter of dental caries remain untreated among the target group (8). Recent evidence by Stormon et al. (2022) indicates an increase of 8% in access to dental services among low-income households through CDBS (7).

It seems that before a conclusion on the evaluation of such schemes and new avenues for policy makers to redesign new interventions or continue the ongoing schemes, it is important to have a big picture of the acts, laws and legislations tabled and released by the parliament as the root and background of such schemes. The Dental Benefits Act 2008 (the Act) which commenced in June 2008 is among one of the critical legislative frameworks for the provision of dental benefits in Australia. The Act has had four reviews over the previous decade. The first review which was tabled in parliament in 2010, mainly focused on the attainment of the purpose and the administration of the Act related to the MTDP. The second review in 2012, concentrated on the operations and administration of the Act related to the MTDP. The third review led to the closure of the MTDP and the establishment of the CDBS and finally, the

fourth review in 2019, considered the operation and administration of the Act, in relation to the CDBS (9).

Being successful with the purpose of implementing such an Act via the stated schemes is not only related to the clarity, power, coverage, and legal and administrative aspects of establishing the rules by the government which are labelled as constitutional rules but also depends on both collective rules and operational rules. In other words, constitutional rules[1], as defined by the government, can affect the formal and informal aspects of collective rules. Collective rules are set by the community groups and can affect the operational rules via their impacts on the demand and supply side of the oral health market including community groups, religious groups, women or other vulnerable parties on the demand side and the professional groups (oral healthcare providers), government and local councils on the supply side. The operational rule is where individuals (users and providers) make choices and where rules-in-form (de jure rules)[2] transform into rules-in-use (de facto rules)[3] (10). Many determinant factors can be considered from the users' perspective while the market forces defined by demand and supply rules can also be influential in day-to-day decisions of the population to utilise the oral health services. These are all considered as operational rules that can be affected directly by collective rules or both directly and indirectly by constitutional rules. Context including this rule in use, socioeconomic and geographic attributes, can affect the whole. Figure-1 illustrates this conceptual framework.

In order to make an understandable and recognisable language between politicians, policy makers and healthcare providers in the area of oral health, we plan to review the content of the Act and its related reviews, considering the government not only as a singular entity and steward of the community`s health but also as the enforcer or the body that implements the rules directed by the acts and at the same time organised by authorities and informal groups and stakeholders (10). Such a framework can present a rational combination among the directions of the acts set by politicians and the governors' rules in oral health. So, this study aimed to analyse the content of the Act 2008 to determine how the Dental Benefits Act encourages Australian families to seek and utilise oral health services.

Method

This qualitative narrative document analysis, applying a framework analysis approach, was conducted in 2022. The full content of the Australian Dental Benefits Act 2008 and the related reviews were considered as the qualitative data. As mentioned by two of the qualitative experts Gupta and Krippendorf, official governmental documents, guidelines and directives, programs, policies and periodic reports, and the content of laws and legislations can be analysed for their contents as a rich qualitative source of data (11,12).

Data collection

Data was collected by searching the formal websites for documents related to the Australian Dental Benefits Act. The whole content of the Act as well as the four versions of its revision were retrieved as the public free documents. The eligibility of the retrieved documents then was investigated and approved according to the suggested criteria by Scott, including authenticity, credibility, representativeness, and meaningfulness of the data (13).

[1] Constitutional rules are based on approved formative constitutions related to fundamental principles of the government`s authority which define the interpretation and application of the powers, rights, and freedoms.
[2] de jure rules (rules-in-form) describe those practices that are legally recognised, regardless of whether the practice exists in reality
[3] de facto rules (rules-in-use) describe those practices that exist in reality, even though they are not officially recognised by laws.

Data Analysis

After retrieving the data and inclusion of the documents according to Scott criteria, we have used the procedure suggested by Gale et al. (2013) for framework analysis including the following seven-steps (14):

- *Transcription*: This first step was not actually done due to the existence of a portal document format (pdf) of the Acts files. So, the content of the files was saved in a word format with large margins and sufficient line spacing for later coding and note taking which occurred in the following steps.
- *Familiarisation*: In order to be familiar with the content of the Acts, all the files were read several times line by line. All the impressions or important issues were noted in the margins of the pages.
- *Coding*: at this point, after familiarisation with the whole texts, the parts related to the research question were highlighted and a paraphrase or label was selected which can describe well the interpretation of the text. These labels demonstrated the initial codes. This was an open coding inductive approach with the aim of summarising the whole data towards the main concept.
- *Developing a working analytical framework*: In this step the initial codes extracted from the content of the Act by one of the researchers (RI) was reviewed by the team and the code labels were discussed, compared, and agreed to by the team. Then the initial codes were categorised to make the final codes apply to a tree diagram to develop an initial analytical framework.
- *Applying the analytical framework*: MAX QDA version 10 was used in this step as a Computer Assisted Qualitative Data Analysis Software to determine the relationships among the initial codes, final codes, and upper-level categories. These categories first create the sub-themes and synthesised the sub-themes to achieve the main themes.
- *Charting data into the framework matrix*: To integrate the inductive open coding approach with a deductive conceptual framework which best fits the emerging concepts, at this stage the triangle of rules proposed by Abimbola (2020) was applied (10). Then the data was charted into a table based on the developed framework (Figure 1).
- *Interpreting the data*: In this last step, the developed framework including the main themes and related sub-themes were described and interpreted considering the comparison among the prior concepts and the new ones emerging from the data.

Data robustness and trustworthiness

Four criteria suggested by Lincoln and Guba were applied to ensure data analysis robustness and trustworthiness including credibility, transferability, dependability, and confirmability (15). To achieve credibility, prolonged engagement with the data occurred through a familiarisation process. The coding process was initially done by RI and NM and finalised by PB through a consensus meeting. An external check was also done by a qualitative research expert to check the whole research process, which was followed by a debriefing session. Presenting a thick description of the findings was then implemented to achieve transferability. To ensure dependability, the whole research process was documented clearly in details and with a logical and traceable manner. And finally, to get confirmability, the interpretation of the findings was described objectively and neutrally to show that all the

findings were derived from the initial data. Furthermore, the reflexivity of the qualitative analysis was assured by the research team collaborating with the qualitative research experts.

Ethical Considerations

Data was analysed by two of the authors (RI and PB) with no conflict of interest against the topic. Ethics clearance was not required.

Results

The content of dental benefits Act 2008 provides directions on all three categories of operational, collective, and constitutional rules. Tables 1 to 3 demonstrate the main themes and sub-themes identified from analysing the Act content in each category of the rules.

Operational rules

Table 1 illustrates three main themes and ten related sub-themes in the category of operational rules. These main themes include consequences of the rules governing how the ~~oral and~~ dental services are used and provided, respond to collective rules as the norms for oral and dental service use and delivery, and respond to regulation on service use and delivery.

According to the results, the content of Act 2008 can either respond to collective rules and norms for service use and delivery and at the same time make a response to regulation on service use and delivery (Table 1). In other words, the content of the Act summarises the advertising norms and other promotion strategies as a marketing mix for improving the utilisation of oral health services and promoting oral health behaviours among particular and vulnerable groups. This can be a defined mechanism of responding to collective and norms by the community to deliver/provide and utilise oral health services. At the same time, the Act summarises the concept of requirements for utilisation of ~~oral~~ services by eligible groups, rate of utilisation and provision of oral health services based on the population strata, financial mechanism, and geographical access as well as legal facilitators and barriers for service delivery, provision, and utilisation. This category of the rules also indicates the consequences of the rules governing how the service is used and provided. Two consequences of the rules can be considered from the content of the Act as sub-themes including general rates of use and delivery of services as well as utilisation and service delivery rate based on specific strata/ geographical areas and public and private sectors.

Insert Table 1- The main and sub-themes related to operational rules

Collective rules

Similarly, Table 2 demonstrates the main theme and sub-themes related to the category of collective rules. The emerged main theme here was "forming the level of the constitution to the enforcement of collective rules" which includes two sub-themes. 'Transforming and adapting the rules from the constitutional level' and 'making the mechanism for monitoring and enforcement of collective rules' are the two sub-themes derived from the Act content to make and form the level of the constitution to the enforcement of collective rules.

Insert Table 2- The main and sub-themes related to collective rules

Constitutional rules

And finally, Table 3 presents a related theme and two sub-themes in the category of constitutional rules. "Legitimising the role of collective level of governance" as the main theme includes two sub-themes of "power reference and steward authority" and "rules and plans: creation, modification, enforcement, and external monitoring". According to table 3, it can be clarified that power reference and steward authority by the Ministry of Health at the national level and authorisation of the states along with creation, modification, enforcement, and external monitoring of the rules and plans can legitimise the role at the collective level of

governance to the constitutional rules at the politician's macro level. This process of creating, modification, enforcing and monitoring of the rules and plans can be implemented and organised by the direct and indirect feedback from the community and service users and as a result of formulating, implementing and evaluating the rules and legislation amendment.

Insert Table 3- The main and sub-themes related to constitutional rules

Considering the results of the present document analysis, politicians and policy makers at the macro and government level can affect all three constitutional, collective, and operational rules. People and families and oral health providers could then make change, monitor, and enforce formal and informal rules which can be in each of the three categories of operational, collective, and constitutional rules. These three categories of rules can then affect their actions, decisions, and relations (Figure 2).

Insert Figure 2- The framework of rules directed from Act 2008

As fi[illegible]e 2 illustrates, operational rules at the level of oral health providers, including dental specialists and the population as the end users of oral health services can be demonstrated as rules in use in a mutual interaction with collective and the constitutional rules. The consequence of governing the rules at the community level can easily define how the oral health services are really provided and utilised. It is clear that the response is sent to the government level for better regulation of oral health service delivery and utilisation, then this is followed with an interaction and advocacy with the diverse range of stakeholders, collaboration across disciplines and capacity to establish, build, and sustain intersectoral and interdisciplinary partnerships with the community groups, non-government sectors, councils, and communities. In this way the rules can be transformed, adopted, monitored, and enforced. Another mechanism of response is occurring from the providers' and users' level and the operational rules to community groups and stakeholders via advertising and promoting the utilisation and provision of oral health services.

Discussion

This study is conducted to determine how the content of the Dental Benefits Act 2008 can help the utilisation of oral health services via two schemes of MTDP and CDBS that the Act mainly focused on during that time. The significance of such an analysis becomes more notifiable and highlighted considering that according to the Australian Institute for Health and Welfare, some groups of the population are at greater risk of poorer oral health, included among them are those with ~~low income or~~ lower socioeconomic status, Australian Aboriginal and Torres Strait Islande[illegible]hose who live in regional and remote areas and those who need additional or special health care needs (16).

To achieve the optimal consequences of oral health services provision and utilisation, the Act pays attention to a dual mechanism of advertising norms and promoting strategies as a marketing mix for oral health services utilisation and at the same time defining the target group via eligibility requirement setting, rates of oral health services utilisation and provision based on geographical area / specific strata, financial mechanisms, and the types of bills. Although it seems that the dual mechanism can be considered as the response to collective rules and regulatory and constitutional rules towards the community and stakeholders and the government, it still needs to concentrate on the other significant factors that could help increase the utilisation of oral health services among vulnerable groups. The evidence from a retrospective utilisation rate of CDBS as one of the emphasised schemes by the Act also shows

that the rate of utilisation during 2014 and 2015 was low and varied among Australian states and territories (17). In contrast with the low rate of utilisation it is important to mention that according to Putri (2020) preventive services were recognised as the most utilised services among the eligible population during 2014-2015 (17). Such evidence can highlight the gap that although the Act includes the concept of advertisement and promotion as the marketing mix for the schemes, attention was not paid to the concept of social marketing as well in order to promote oral health behaviours. At the same time, in contrast with the inclusion of eligibility requirements for the target groups in the Act, according to the findings of Orr et al. (2021) some lower levels of preventive services utilisation are obvious among Aboriginal children as one of the most eligible and vulnerable groups (18).

The Act has considered two concepts of power reference and steward authority and creation, modification, enforcement, and external monitoring of the rules and plans for legitimising the role of collective level of governance. In this category, although the concepts of people's communication channels and people`s feedback about the schemes are considered, finding of Nguyen et al. (2020) have shown the issue seems more complex so that those mothers in the lower socioeconomic status group, or those who have experienced any kind of depression or with lower levels of mental health, as well as, the mothers with a type of health behavioural problem like smoking or even poor oral health behaviours, had the lower tendency to claim the available benefits of CDBS for their children (6) so it would be important to find the mechanism for weighting the feedback or finding some more robust channels and real feedback of utilisation of the services by the Australian families.

Another overlooked point that needs more attention in the potential revisions of the Act is to focus more on the coverage of the services highlighted in the Act. For instance, although the present results show that monitoring, transforming, adopting and enforcement of the rules is important to translate them into practice by the end users, evidence implies that some types of preventive services provisions by an alternative workforce are not supported by the fourth revision of the Act such as fluoride varnish items provided by dental assistants because there was not any defined mechanism for receiving and reviewing the changes and revisions via the Act (18). Similarly, for those Indigenous population groups who use CDBS voucher, two concerns are mentioned. Firstly, the variety of service provision based on different locations that led to a poor coverage of only a bit more than 20% of the concession card holders and secondly the exclusion of many services like orthodontics from CDBS and the need for such services to be undertaken by the private sector (19). So, as the main concentration of the Act and the CDBS is on preventive schemes, it would be more cost-effective and valuable to redesign a basic benefit package to include all the probable cost-effective services according to the needs of the eligible population, as well as their unmet needs, in the next revisions of the Dental Benefit Act (2008). Also, from the coverage perspective, there should be a new policy dialogue to identify strategies for increasing the utilisation of the services particularly during and after COVID-19 era. According to Hopcraft and Farmer (2020), from a retrospective analysis of Medicare data on CDBS utilisation, a large decline was observed in the provision of preventive and diagnostic services during the pandemic in comparison with the utilisation of endodontic and oral surgery services (20).

This study attempted to integrate the perspective of politicians with those of policy makers to reconsider the role and significance of the rules based on the triple collaborations among oral

health users and oral service providers, the community, and the stakeholders as well as the government. However, the study was accompanied with some limitations as follows: the national document analysis of the Acts and legislations would have been enhanced if it were triangulated with an in-depth qualitative data collection (and analysis) from the perspective and experiences of service users and providers, as well as the inclusion of quantitative statistics for the utilisation rate of the schemes. Undertaking a mix-method study for a holistic view prior to the further revision of the Act or probable upcoming schemes are recommended.

Conclusion

Utilisation of the oral health services by the population is a strategy considered by Australian health system as a way of achieving, as a final goal, optimum oral health for its population. For this purpose, coordination and integration of the laws and legislations at the Parliament and politician level with those applied policies, interventions, schemes and regulations by the government and policy makers is necessary. The Dental Benefit Act 2008 as a foundation for MTDB and CDBS is among the Australian legislations which tries to consider all the operational, collective, and constitutional rules with a mutual relationship among the population as the end users of oral services, oral health providers, community and stakeholders and the government and oral health policymakers. However, a comprehensive attention is still needed in the future revisions of the Act according to the contextual factors, socioeconomic and geographical attributes of the population for better implementation of de facto rules and more effective outcomes of the interventions.

Abbreviations

WHO: World Health

CDDS: Chronic Disease Dental Scheme

MTDP: Medicare Teen Dental Plan

CDBS: The Child Dental Benefits Schedule

Declarations

Ethics approval and consent to participate

All the methods were performed in accordance with the relevant guidelines and regulation.

Consent for publication

Not applicable.

Availability of data and materials

The datasets used and/or analysed during the current study are available from the corresponding author on reasonable request.

Competing interests

The authors declare that they have no competing interests.

Funding

There was no funding.

Authors' contributions

PB has designed the study and analysed the data, RI has contributed in data analysis and article drafting, NM and DH have contributed in finalizing the manuscript, AG and HC have contributed in developing the conceptual framework and LD has supervised the whole study.

Acknowledgements

Not applicable.

Table 1- The main and sub-themes related to operational rules

Theme	Sub theme	Final code
Consequences of the rules governing how the service is used and provided	**General rates of use and delivery of services**	Low CDBS[a] usage rate as a reflection of poor promotion
		Increase the number of active providers, service recipients, benefits, and CDBS performance indicators
		Low rate MTDP[b] plan uses in eligible teenagers and lack of continuous increase rate in using vouchers
		MTDP: Issuance of coupons and services per coupon per year for eligible individuals
		MTDP overall success, based on goals and performance indicators
		MTDP: weakness in Actual benefits paid, utilisation target, actual utilisation allocation
	**Utilisation and service delivery rate based on specific strata/ geographical areas and public and private sectors**	CDBS: The similarity of the usage rate in major cities and regional areas, sharp decrease in the usage rate in remote areas
		CDBS: Lowest service usage in SA and Tasmania and highest in Northern Territory and WA
		CDBS: A reasonable proportion between the eligible population and the rate of service use in each SEIFA[c]decile
		CDBS: Lower use rates for aboriginal children than non- aboriginal children, despite higher population eligibility
		Injury to needy patients, especially distant children with irregular visitation patterns due to the exclusion of CDBS for in-hospital dental services
		CDBS: Revealing private sector dominance in service delivery in Australia-wide
		CDBS: Uncertainty in access to services due to administrative and financial problems in public sector
		CDBS: The need to expand the private sector in NSW and the public sector in SA and Tasmania, and cooperation between states to understand the different use rates and remove restrictions
		MTDP: Similarity in bulk billing rates in major cities, inner and outer areas, but higher bulk billing rates in remote areas, and higher uptake rates in more affluent areas due to more providers
Respond to collective rules: norms for service use and delivery	**Advertising norms affecting the use and delivery of services**	CDBS: The need to increase advertising to clarify the importance of oral health in children
		CDBS: Use of other advertising methods (campaign, brochures, radio, video in public centers, dental offices, ethnic press, school newsletters, social media) along with eligibility notifications
		Need to revise the eligibility notifications to ensure better understanding of recipients and more attractiveness, poor promotion, and the need for more serious marketing in CDBS
		MTDP: Managing family expectations using appropriate communication materials and easy-to-understand content to increase the rate of absorption and use
		MTDP: Public advertising of plan through brochures, websites, social media at the beginning and throughout the year
	**Promoting as a marketing mix for specific groups**	the need to focus promotion among needy and special groups (communication channels, Follow-up letter)
		MTDP: Advertisements through letters to dentists and families with qualified teenagers, advertisements focused on specific groups (school newsletters, ethnic press, community radio)
		Focus on promoting MTDP amongst at-risk groups by community leaders, charity groups, and dentists
		MTDP: Clarification and explanation of the value of vouchers for high-risk or impassable groups
		Eligibility notifications as the most important method of promotion,
		The need to expedite the issuance of vouchers and improve its branding as the strongest communication element
Respond to regulation on service use and delivery	**Eligibility Requirements**	Condition for using Medicare Teen Dental Plan, eligibility in terms of age (12-17) and mean test
		MTDP: Interpretation of the means test to determine of eligible teenagers12-17years old in "FTB-A"[d] recipient families or "Youth Allowance or Abstudy" recipient families
		Condition for using CDBS, eligibility in terms of age or member of the eliminated needy groups but recommendations to reduce the age of eligibility due to the importance of dentistry in one to two years
	**Service utilisation and provision subject to eligibility of individuals**	MTDP: Clarification of the issue of not providing voucher physically during the visit, no need for physical voucher, verification of eligibility by contacting Medicare by dentists
		CDBS: check eligibility notifications by phone and net/no need for physical form, adequate justification of dentists and emphasis on the need to check some items before providing the service (eligibility, number of benefits left) via SMS
		Step-down fees for certain services more than one service per day in CDBS (fissure sealants, five extraction items)
		CDBS: the evidence of need, clinical efficacy, and safety (introducing new items)
		Restrictions aimed at providing more appropriate services and benefits to patients

	**Legal scope of service delivery and legal restrictions**	Exclusion of CDBS dental benefits for in-hospital dental services, the need to remove limitation to prevent harm to needy patients using the Commonwealth and state budget allocated for this issue.
	**Rate of service utilisation and provision based on geographical area / specific strata**	Calculation of Accessibility/Remoteness index (including eligibility and utilisation) based on geographical standards, the residence of many eligible people in major cities and remote areas
		Calculation of SEIFA index (based on geographical areas), with the aim of determining the eligible children with the highest financial needs in each state and comparison of states
		Identify areas based on target population and utilisation rate, target increasing utilisation rates in the Northern Territory
		Identify the overall rate of use of CDBS and the number of services provided by the public and private sectors based on geographical areas,
		Identification of aboriginal and Torres Strait Islander children through Medicare Indigenous Voluntary Identifier, comparison of eligibility and use between indigenous and non-indigenous
		sharp decrease in the usage rate in remote areas due to lack of dentist, lack of fluoridated water supply and other factors
	**Service provision and utilisation rate based on the type of bill**	Two types of claim models for CDBS: bulk billing with potential implications for accessing services & patient billing as a deterrent for certain patient groups
		Payment of plan benefits in two ways, a large part in the form of bulk bill, and the rest in the form of private bill
	**Service provision and utilisation subject to financial and treatment consent**	Complete and register the signed form of financial & treatment consent of eligible patients: 'Bulk Billing Patient Consent Form' on the first day, and 'Non-Bulk Billing Patient Consent Form' per visit
		Exemption of the provider from obtaining informed financial consent in certain circumstances according to the Ministerial Guidelines

[a] CDBS: Children Dental Benefit Schemes
[b] MTDP: Medicare Teen Dental Plan
[c] SEIFA: Socio-economic indices for areas
[d] FTB-A: Family Tax Benefit-Part A

Table 2- The main and sub-themes related to collective rules

Theme	Sub theme	Final code
From the level of the constitution to the enforcement of collective rules	**Transform and adopt rules from constitutional level**	Coordination of the DBA[a] 2008 and the HIA[b] 1973 on penalty & recovery arrangements, and amendments, but difference between the two in the index of access to benefits
		Amendment (2018) aimed at aligning DBA 2008 and HIA 1973 on alignment of administrative arrangements, strengthening debt recovery (bulk-billing), the garnishee arrangements (not bulk-billing)
		DBA, a framework for determining payments, entitlement, protection & disclosure information, issuance of vouchers, provisions on assignment of benefit & providing false information
		Rules 2008 /2009 relating to the MTDP[c] operating framework and Rules 2013/2014 relating to the CDBS[d] operating framework
		CDBS, as determinant of the eligible providers/children and services, how to advertise and promote the program, and conditions for payment (modeling CDBS payable benefits based on ADA[e])
		CDBS's main purpose: provide payable benefits for 'basic dental' services, identify CDBS items in the ADA dictionary using the additional two-digit prefix
		The need to create an alternative process to reduce the administrative burden on access to benefits in case of ambiguity the patient's eligibility when receiving service (Clarification of extenuating circumstances in the Ministerial Guideline)
		Defects in the mean test method, eliminating some needy teens based on this test, and need for group consultation with relevant organisations to expand the eligible conditions of teenagers with the aim of covering the needy groups eliminated by the means test
		Administrative and eligibility requirements in the Rules (2008-2009); Designation of dental providers, eligible people for a voucher, duration and conditions required to issue the vouchers, information required to complete the account form, conditions without the need to issue a coupon
		Eligible people & dental providers / accounts/invoices / Rules regarding the payment time of each service / Patient consent form rule in consent to treatment/ patient consent form rule in financial consent/ Requirement to keep clinical records (4years)
		better panel understanding of the social and geographical conditions of vouchers recipients and users using SEIFA[f] index analysis, data on vouchers and services, and data related to unallocated groups, and data on receipt and use of vouchers based on bill type and geographical area
		Draw charts and bills based on the "Australian Schedule of Dental Services and Glossary" as a generally coding system, and assign a three-digit code to clinical items or procedures in the ADA schedule
	**Monitoring and enforcement of collective rules**	Funds relating to the payment of DBA through a special appropriation
		Dental Benefits payable for annual preventive dental check-ups through a dedicated unit number
		Dedicated use of the 'voucher' terminology in MTDP rules, but removing it in advertising and replacing it with notification of eligibility
		CDBS: Voucher validity from the time of issue until the end of the calendar year, and applying retrospective eligibility to covering expenses before issuing coupons in the same year, but existence of administrative challenges in this method
		Financing under special appropriation in CDBS, and the upward trend of its expenditure from the beginning of the plan
		CDBS: Payment of plan benefits based on the claim processing date
		MTDP: Providing preventive dental checkups in the form of bulk bill in public dental clinics
		Incremental rate of projected administered budget under Medicare Teen Dental Plan (2008-2015)
		Providing the eligible verification information by Centrelink/DVA[g], matching it with Medicare information for vouchers issuance and sending bulk vouchers at the beginning of the year and sending new vouchers monthly from March via mail-out
		CDBS: providing eligibility information by Centrelink/DVA, matching with Medicare information for issuance of notification of eligibility, sending eligibility notifications letter to families, teenagers, and Approved Care Organisations at the beginning of the year and every two weeks (mail-out or myGov)
		Behavior changes in teenagers and improvement of oral health consequences due to proper management of MTDP and problems like lack of motivation of dentists due to fixed cost structure
		Systemic problems for dentists, inability to analyse data, patient uncertainty about services received during the examination, other problems (shifting between public and private sector, the plan's lack of responsibility for ongoing treatment, the need to ensure non-examination by students)
		CDBS: The requirement to obtain a second "signature" from the guardian for electronic billing/solve the issues by amending bulk billing payment service rules and redesigning the consent

[a] DBA: Dental Benefits Act [b] HIA: Health Insurance Act [c] CDBS: Children Dental Benefit Schemes [d] MTDP: Medicare Teen Dental Plan[e] ADA: Australian Dental Association[f] SEIFA: Socio-economic indices for areas [g] DVA: Department of Veterans` Affairs

Table 3- The main and sub-themes related to constitutional rules

Theme	Sub theme	Final code
Legitimize the role of collective level of governance	Power reference and steward authority	The power of the Minister of Health to determine the Dental Benefits Rules using the legislative instruments to create an operational framework for the payment of dental benefits under this Act
		CDBS[a] management by the Department of Human Services using information about eligibility, providing eligibility information by Centrelink/DVA[b], matching them with Medicare information, issuance of notification of eligibility based on the degree of compliance
		Authorisation of states to provide access to CDBS services by Minister for Health (granting access or re-access), with the aim of providing services in areas without private sector activity and permanent access to CDBS with a long-term plan
		MTDP[c] plan management by the Department of Human Services using information about eligibility
		MTDP: Introduction of "RPDs"[d] by the states, preventive checkups by them, payment of RPDs -related benefits to a state bank account with income tax deduction and possibility of providing dental services by one state on behalf of another state
	Rules and plans: creation, modification, enforcement, and external monitoring	Designing the DBA[e] 2008 based on the HIA[f] 1973, and introducing the Health Legislation Amendment (2018) to further align HIA 1973 and DBA 2008,
		Dental Benefits Rules 2008-2009 with the aim of determining the requirements of the MTDP and to establish a new DBS[g]
		The ACT 2008 competence in designing a framework for the payment of dental benefits, its competence in the proper administration of the Medicare Teen Dental Plan, and finally the success of this plan (Examining these issues as the task of the committee (panel)
		Limited implementation of the DBA, to date (2019)
		MTDP role through the provision of preventive services and examinations in the form of vouchers (up to a benefit of $ 150 & the increasing rate of benefits over time) in public and private centers
		Replacing the 2009 Rules with the 2013 Rules, thus ending the MTDP and starting the CDBS, with the increase in the acceptable age range, the number of eligible people, the range of services, higher profit margins, and the use of specific item numbers for each service
		Creating CDBS, for eligible children aged 2-17 and benefit level of $ 1,000 and usable for up to two years for essential preventive services to improve oral health by creating habits in the long run, targeting Commonwealth expenditure on Children's oral health, building a national system for pediatric dentistry
		Modeling of payable benefits in CDBS plan, based on ADA[h]
		Patterned from Chronic Disease Dental Scheme, but the need to adapt CDBS based on Veterans' Affairs 'step-down fees' model to improve clinical effectiveness and address problems (Especially by adopting the "per quarter" model)
		People's communication channels for feedback about MTDP: ministerial correspondence and direct contact
		The most common issues based on people's feedback from MTDP: benefit level (Money value) for preventive checkup, ability to receive more comprehensive service at the same price based on the interview, benefits payable only for one preventative checkup

[a] CDBS: Children Dental Benefit Schemes
[b] DVA: Department of Veterans` Affairs
[c] MTDP: Medicare Teen Dental Plan
[d] RPD: Representative Public Dentists
[e] DBA: Dental Benefits Act
[f] HIA: Health Insurance Act
[g] DBS: Dental Benefits Scheme
[h] ADA: Australian Dental Association

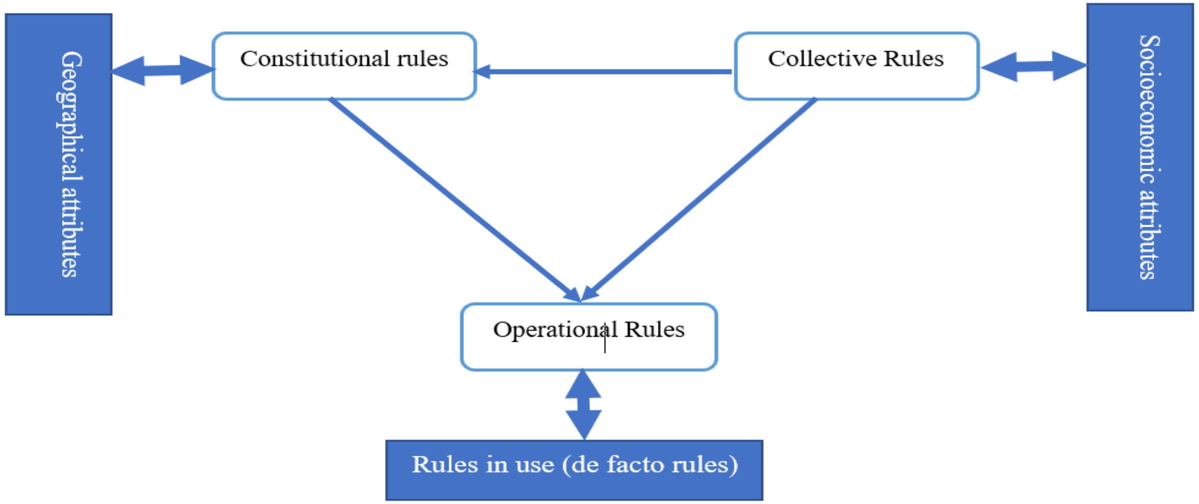

Figure 1- Conceptual framework of the analysis

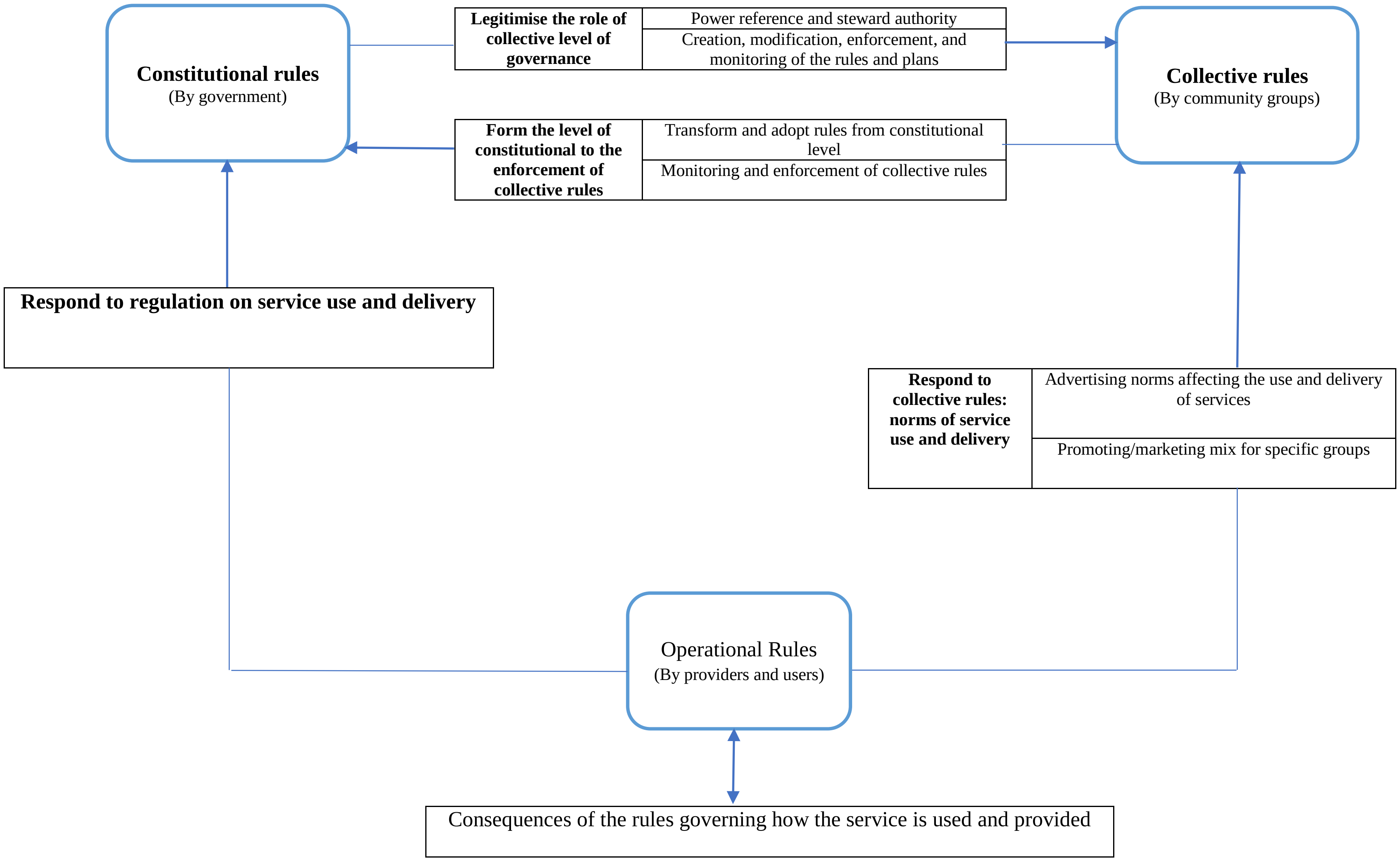
Constitutional rules
(By government)
Legitimise the role of collective level of governance
Power reference and steward authority
Creation, modification, enforcement, and monitoring of the rules and plans
Collective rules
(By community groups)
Form the level of constitutional to the enforcement of collective rules
Transform and adopt rules from constitutional level
Monitoring and enforcement of collective rules
Respond to regulation on service use and delivery
Respond to collective rules: norms of service use and delivery
Advertising norms affecting the use and delivery of services
Promoting/marketing mix for specific groups
Operational Rules
(By providers and users)
Consequences of the rules governing how the service is used and provided

Figure 2- The framework of rules directed from Act 2008

References

1- Peres MA, Macpherson LMD, Weyant RJ, Daly B, Venturelli R, Mathur MR, Listl S, Celeste RK, Guarnizo-Herreño CC, Kearns C, Benzian H, Allison P, Watt RG. Oral diseases: a global public health challenge. Lancet. 2019 Jul 20;394(10194):249-260.
2- Spanemberg JC, Cardoso JA, Slob EMGB, López-López J. Quality of life related to oral health and its impact in adults. J Stomatol Oral Maxillofac Surg. 2019 Jun;120(3):234-239.
3- Petersen PE. World Health Organization global policy for improvement of oral health-world health assembly 2007. Int Dent J. 2008;58(3):115–21.
4- Mohammadpour M, Bastani P, Brennan D, Ghanbarzadegan A, Bahmaei J. Oral health policymaking challenges in Iran: a qualitative approach. BMC Oral Health. 2020 Jun 1;20(1):158.
5- Policies and interventions to improve oral health, NICE pathway, 2018. Available from: http://pathways.nice.org.uk/pathways/oral-health-improvement-for-local-authorities-and-their-partners
6- Nguyen TM, Tonmukayakul U, Calache H. A dental workforce strategy to make Australian public dental services more efficient. Hum Resour Health. 2019 May 30;17(1):37.
7- Stormon N, Do L, Sexton C. Has the Child Dental Benefits Schedule improved access to dental care for Australian children? Health Soc Care Community. 2022 Mar 25.
8- Do L, Spencer AJ, editors. Oral health of Australian children: the National Child Oral Health Study 2012–14. Adelaide: University of Adelaide Press; 2016.
9- Australian Government, Department of Health. Available from: https://www1.health.gov.au/internet/main/publishing.nsf/Content/Dental_Report_on_the_Review_of_the_Dental_Benefits_Act_2008
10- Abimbola S. Health system governance: a triangle of rules. BMJ Glob Health. 2020 Aug;5(8):e003598.
11- Gupta RK, Awasthy R. Qualitative research in management methods and experiences. SAGE Publication Ltd, 2015.
12- Krippendorf KH. Content Analysis: An Introduction to its Methodology London, Sage,3rd ed,2012
13- Tashakkori A, Teddlie C. Handbook of mixed methods in Social & Behavioral Research. SAGE Ltd. 2003:297–320.
14- Gale NK, Heath G, Cameron E, Rashid S, Redwood S. Using the framework method for the analysis of qualitative data in multi-disciplinary health research. BMC Med Res Methodol. 2013 Sep 18;13:117.
15- Nowell LS, Norris JM, White DE, Moules NJ. Thematic Analysis: Striving to Meet the Trustworthiness Criteria. International Journal of Qualitative Methods. 2017; 16: 1–13
16- Oral health and dental care in Australia. Available from: https://www.aihw.gov.au/reports/dental-oral-health/oral-health-and-dental-care-in-australia/contents/introduction
17- Putri DE, Kruger E, Tennant M. Retrospective analysis of utilisation of the Australian Child Dental Benefit Scheme. Aust Health Rev. 2020 Apr;44(2):304-309.
18- Orr N, Gwynne K, Sohn W, Skinner J. Inequalities in the utilisation of the Child Dental Benefits Schedule between Aboriginal and non-Aboriginal children. Aust Health Rev. 2021 Jun;45(3):274-280.
19- Butten K, Johnson NW, Hall KK, Toombs M, King N, O'Grady KF. Impact of oral health on Australian urban Aboriginal and Torres Strait Islander families: a qualitative study. Int J Equity Health. 2019 Feb 18;18(1):34.
20- Hopcraft M, Farmer G. Impact of COVID-19 on the provision of paediatric dental care: Analysis of the Australian Child Dental Benefits Schedule. Community Dent Oral Epidemiol. 2021 Aug;49(4):369-376.
